# Stabilizing the Sealing Performance of EPDM by the Incorporation of a ZIF-8 Network

**DOI:** 10.3390/polym18070874

**Published:** 2026-04-02

**Authors:** Jiahui Chen, Qian Peng, Huadong Liu, Xingtao Xiao, Xiaotao Fu, Hanlin Wen, Zhicheng Huang, Fangqiang Wang, Xiaoliang Zeng

**Affiliations:** 1 Electric Power Research Institute, State Grid Sichuan Electric Power Company, Chengdu 610041, China; pq8324@163.com (Q.P.); ilhd123456@163.com (H.L.); hxc20170113@163.com (Z.H.); wangfq114@126.com (F.W.); zengxiaoliangscu@126.com (X.Z.); 2 Direct Current Branch, State Grid Sichuan Electric Power Company, Chengdu 610000, China; xiaoxt2030@163.com (X.X.); wasl0n@163.com (X.F.); 15828331244@163.com (H.W.)

**Keywords:** ZIF-8, EPDM, mechanical performance, aging resistance

## Abstract

Ethylene–propylene–diene monomer rubber (EPDM) is commonly used as a gas-tight sealing material in electrical equipment. Factors such as media exposure, thermal oxidative stress, and abrasion frequently cause deterioration of EPDM’s mechanical properties, significantly compromising the reliability of electrical equipment. Traditional activator ZnO provides limited enhancement to the properties of EPDM. The reaction between Zn^2+^ on the surface of zinc oxide interacts with the accelerator during curing of rubber, forming zinc chelates, which interact with sulfur to form zinc polysulfide complexes. But the release of zinc complexes has adverse effects on humans and ecosystems. To reduce ZnO usage and further improve the performance of EPDM in terms of mechanical properties and aging resistance, zeolitic imidazolate framework-8 (ZIF-8) is developed as a multifunctional additive in this work. Mechanical testing demonstrates that the incorporation of ZIF-8 enhances the mechanical performance and resistance to thermal oxidative aging of EPDM. Crosslink density testing, FTIR, and XPS show that ZIF-8 promotes the crosslinking reaction during rubber curing, resulting in improved mechanical performance for EPDM. Analysis of crosslinking density testing and SEM images shows that EPDM-ZIF-8 composite exhibits a slower increase in crosslinking density during thermal oxidative aging. TGA results indicate that ZIF-8 enhances the thermal stability of EPDM, which leads to improved aging resistance properties. This study provides new insights for the design and development of rubber composite materials, offering a reliable solution to the challenge of seal failure in electrical equipment.

## 1. Introduction

Rubber holds a unique position in modern science and technology; it exhibits outstanding tack and excellent mechanical performance after curing, making it a commonly selected sealing material [1,2]. To accommodate diverse sealing situations, rubber has evolved from natural rubber into numerous varieties after a long period of development [3,4,5]. Among these, EPDM is frequently employed as a gas-tight sealing material in electrical equipment. In recent years, failures of seals have frequently led to electrical equipment malfunctions and shutdowns in ultra-high-voltage converter stations, which poses a significant challenge to the stable operation of power systems. Modifying rubber to optimize its mechanical properties and aging resistance holds profound significance for maintaining the stable operation of power systems.

Currently, in the global commercial production of rubber, ZnO is extensively used for curing. ZnO is regarded as the most effective of all activators and exerts a pivotal influence during the incipient phases of curing to attain higher crosslinking densities [6,7]. It does so by enhancing the kinetics of the rubber curing process and facilitating the generation of sulfide crosslinking. The reaction between Zn^2+^ on the surface of zinc oxide interacts with the accelerator during curing of rubber, generating the zinc chelates, which interact with sulfur to form zinc polysulfide complexes [8,9]. Subsequently, these complexes bond with the polymer chain network of rubber to create a crosslinking network, improving the mechanical performance of rubber [10]. The size, crystal structure, and specific surface area of the ZnO particles, as well as their dispersibility in the rubber matrix, significantly restrict the touch and coaction between the ZnO and the accelerators, which results in poor stability of rubber cured with ZnO [11,12]. In addition, release of zinc complexes occurs in processes such as decomposing, recycling, and manufacturing of rubber, with adverse effects on humans and ecosystems. Therefore, it is necessary to find an alternative to ZnO to decrease the quantity of ZnO and further effectively improve the performance of rubber.

Zinc-based ZIF-8 materials exhibit significant potential; the extremely high specific surface area, square sodium topology and multiple potential interaction sites of ZIF-8 provide stronger additive–matrix interface interactions [13,14,15,16]. Therefore, replacing Zn-O with ZIF-8 as an activator represents a potentially effective approach to enhancing the mechanical performance and aging resistance of rubber. Katarzyna et al. [17] investigated the effect of zinc-containing metal–organic frameworks (MOFs) on the vulcanization process of Styrene Butadiene Rubber (SBR); by partially substituting ZnO with MOFs at varying degrees, it was found that a 1:1 ratio of MOFs to ZnO yielded the most favorable activator for vulcanization, resulting in greater enhancement of SBR’s mechanical properties. Tian et al. [18] incorporated ZIF-8, ZIF-11, and ZIF-14 into natural rubber (NR) respectively; by comparing the vulcanization characteristics and mechanical properties of NR with different zinc-containing zeolitic imidazolate frameworks, they demonstrated that ZIF-14 exhibited the most pronounced promotion effect on curing of NR at 130 °C; the promoting effect of ZIF-11 was not significant, while ZIF-8 exerted the greatest positive influence on NR in terms of crosslink density and mechanical properties. Yang et al. [19] substituted ZnO with ZIF-8 in NBR, demonstrating that ZIF-8 particles exhibit stronger interfacial interactions with the NBR matrix, thereby enhancing the material’s resistance to hydrogen blistering. ZIF-8 promotes increased crosslinking density by facilitating uniform dispersion of silica particles, which improves the mechanical performance of Nitrile Butadiene Rubber (NBR). Although previous researchers have conducted extensive studies on the modification of rubber with ZIF materials, the effects of ZIF materials on EPDM and the associated mechanisms remain unclear.

In summary, ZIF-8 shows broad application prospects in the rubber industry, yet its use in EPDM has not been explored. Herein, a simple synthesis method for ZIF-8 is developed, which is suitable for large-scale production. The synthesized ZIF-8 is then used to modify EPDM as an activator in the EPDM curing process, and we analyze its effects on EPDM in terms of mechanical properties and aging resistance and elucidate the underlying mechanisms by which ZIF-8 influences these characteristics.

## 2. Experimental Procedure

### 2.1. Materials

Ethylene–propylene–diene monomer rubber (EPDM) was obtained from Shandong Taikai Co., Ltd., Taian, China. 2-mercaptobenzothiazole (MBT), antioxidant N-(1,3-dimethylbutyl)-N′-phenyl-pphenylenediamine (6PPD), sulfur, silica, stearic acid, and zinc oxide (ZnO) were supplied by Sinopharm Chemical Reagent Co., Ltd., Shanghai, China. The composition of the EPDM/EPDM-ZIF-8 is listed in Table 1.

### 2.2. Accelerated Aging Experiments

Three specimens per group are placed in a 125 °C air-circulating aging chamber for thermal oxidative aging. The specimens are conditioned at room temperature for 18 h before performance testing. Samples are removed from the aging chamber once every 24 h, with a total aging duration of 168 h. The thermal oxygen compression set test is conducted on the samples at 125 °C using a load limiter with an initial compression ratio of 25%.

### 2.3. Transmission Electron Microscopy (TEM)

Transmission electron microscopy (thermoscientific Talos F200X G2, Waltham, MA, USA) is used to explore the micro-morphology of ZIF-8 and ZnO. The samples are observed using the bright-field imaging mode at an accelerating voltage of 80 kV.

### 2.4. Crosslink Density

At ambient temperature, EPDM and EPDM-ZIF-8 are separately immersed in cyclohexane for a swelling period of 72 h. Prior to measuring the swollen mass, the surface of the samples is blotted dry with absorbent paper. The crosslink density is calculated by applying the Flory–Reynner equations, which are presented in Equations (1) and (2).

(1) $$\nu_{e}=\frac{-[V_{r}+\chi V_{r}^{2}+\ln(1-V_{r})]}{\left[V_{s}(V_{r}^{\frac{1}{3}}-V_{r})/2\right]}$$

(2) $$V_{r}=\frac{m_{r}}{m_{r}+m_{s}(\rho_{r}/\rho_{s})}$$

The crosslink density per unit volume is denoted as *ν*_e_ (mol·cm^−3^), and the polymer volume fraction of the swollen sample is represented by *V*_r_. The mass of the rubber is m_r_ (g), while the mass of the solvent in the swollen sample is m_s_ (g). The density of the rubber is *ρ*_r_ (g·cm^−3^), and the density of the solvent (cyclohexane, with a density of 0.779 g·cm^−3^) is *ρ*_s_. The polymer–solvent interaction parameter is *χ* (with a value of 0.321) [20]. The molar volume of the solvent (cyclohexane, having a molar volume of 108.04 cm^3^∙mol^−1^) is V_s_.

### 2.5. X-Ray Photoelectron Spectroscopy (XPS)

X-ray photoelectron spectroscopy (XPS, GENESIS 900, ULVAC-PHI, Chigasaki, Japan) is utilized to characterize EPDM and EPDM-ZIF-8 rubber samples. The aim is to explore the alterations in surface elemental composition and chemical states that occur during curing.

### 2.6. ATR-FTIR Analysis

To explore the alterations in the chemical structures of various EPDM samples, attenuated total reflectance Fourier transform infrared spectroscopy (ATR-FTIR, Spectrum 100, PerkinElmer, Waltham, MA, USA) is employed. The spectra are registered within the wavenumber interval from 4000 to 400 cm^−1^.

### 2.7. Thermogravimetric Analysis (TGA)

The PerkinElmer TGA4000 (PerkinElmer, Waltham, MA, USA) is used to carry out thermogravimetric analyses at three different heating rates (20 °C/min). Samples with a mass ranging from 10 to 15 mg are heated up to 700 °C under a nitrogen flow, with a feed rate of 20 mL/min.

## 3. Results and Discussion

### 3.1. Modification of EPDM

#### 3.1.1. ZIF-8 Preparation

**Figure 1:**
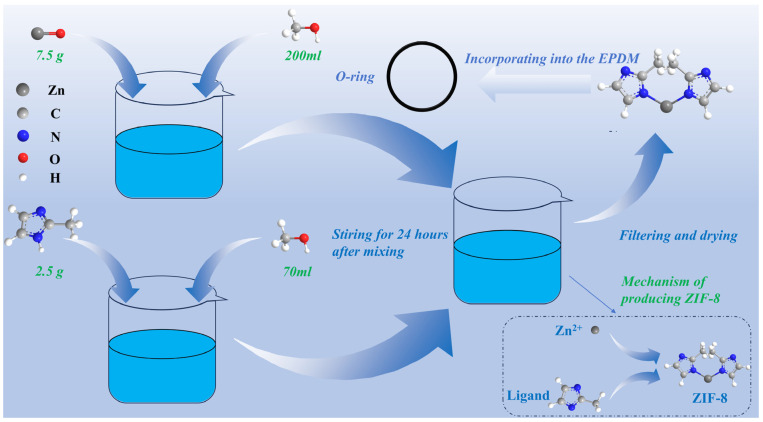
A schematic diagram of ZIF-8 preparation and EPDM modification.

The procedure for preparing ZIF-8 is presented in Figure 1. First, 2.5 g of dimethylimidazole is dissolved in 70 mL of methanol, and 7.5 g of zinc oxide is mixed with 200 mL of methanol to prepare a zinc oxide suspension. The zinc oxide suspension enhances the dispersion of zinc oxide, facilitating the subsequent coordination reaction between Zn^2+^ and MIM^−^ on the surface of the zinc oxide to form ZIF-8 layers. Then, combine the two solutions and stir them for 24 h using a magnetic stirrer. Filter the liquid after stirring for 24 h using a filtration apparatus, then filter again after washing the filtered solids with deionized water. The filtered solids are dried to attain a mixed powder of ZnO and ZIF-8.

#### 3.1.2. ZIF-8 Characterization

**Figure 2:**
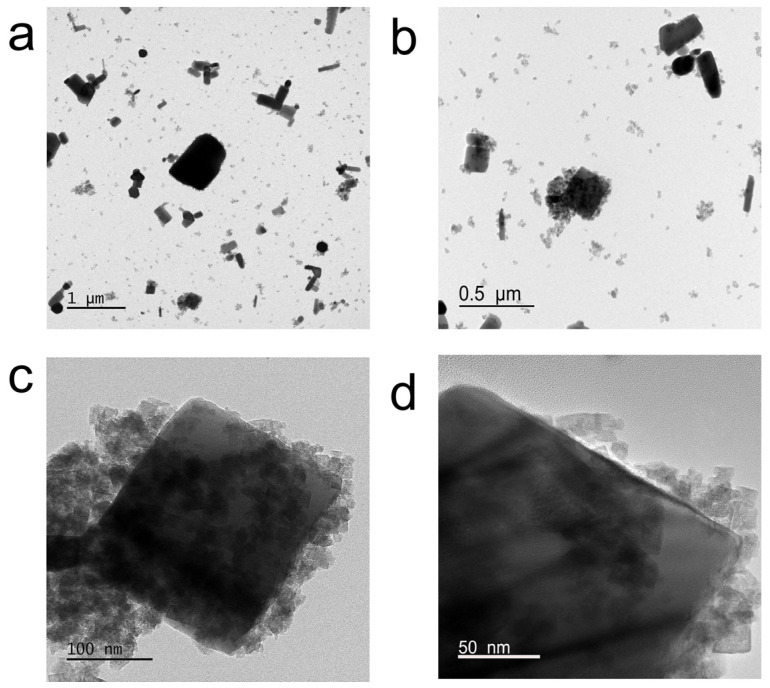
TEM images: (**a**) ZnO; (**b**) ZIF-8 capped ZnO; (**c**,**d**) high-magnification TEM images of ZIF-8 capped ZnO.

To investigate the microstructure of ZIF-8, TEM testing is performed on the prepared solid powder. The TEM image of pure ZnO is shown in Figure 2a, showing that pure ZnO exists as a bulk solid. As observed in Figure 2b–d, the ZnO surface is coated with flocculent material that is identified as ZIF-8. These results indicate that nucleation and crystallization of ZIF-8 primarily occur on the surface of ZnO. Zn^2+^ is released from the surface of zinc oxide when a suspension containing zinc oxide is mixed with a methanol solution of 2-MIM [21]. 2-MIM undergoes ionization to form an MIM^−^ ligand in the methanol solution, and methanol promotes this ionization reaction by forming hydrogen bonds with 2-MIM [22]. The coordination reaction occurs after the generated MIM^−^ ligand encounters the released Zn^2+^, leading to the nucleation of ZIF-8. Nucleation of ZIF-8 occurs, followed by crystal growth through continued surface conversion processes [23]. The reaction mechanism of ZIF-8 is shown as follows:

2-MIM ↔ MIM^−^ + H^+^ (3)

2-MIM + CH_3_OH → MIM^−^ + CH_3_OH_2_^+^ (4)

Zn^2+^ + 2MIM^−^ → Zn(MIM)_2_ (5)

#### 3.1.3. EPDM Mixing and ZIF-8 Incorporation

The prepared mixed powder is added as an additive during the EPDM manufacturing process. Mix EPDM and 6PPD in a mixer for 3 min to prepare the EPDM compound. Then, silica, stearic acid, and MBT are introduced and blended for five minutes at a temperature of 100 °C, while the rotor is spinning at a speed of 40 rotations per minute. The system containing 2 phr of mixed powder with ZIF-8 and ZnO is compared with the system containing 2 phr of ZnO alone, and then sulfur is added and the mixture is stirred for 5 min. Compounds are milled in a twin-roll mixer for 3 min to promote additive dispersion. The compounds are injected into the mold and then vulcanized at 140 °C for 30 min under 15 MPa. The finished O-rings have an inner diameter of 189 mm and a cross-sectional diameter of 3.55 mm.

### 3.2. Mechanical Performance and Aging

**Figure 3:**
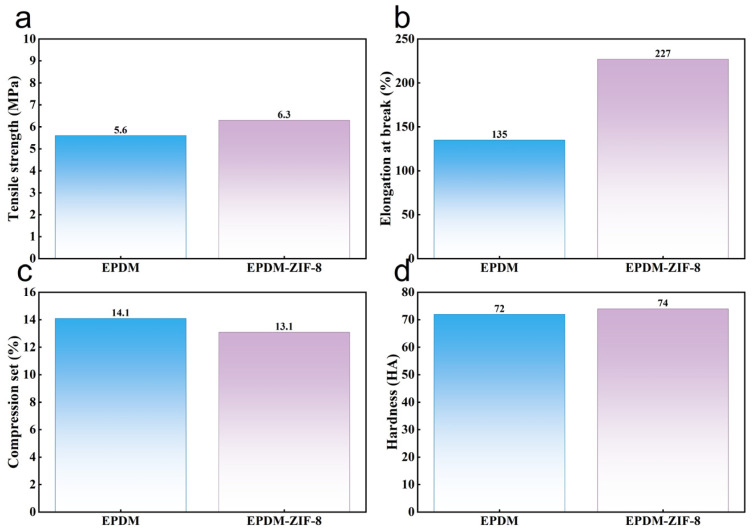
Comparison of mechanical performance before and after ZIF-8 modification: (**a**) tensile strength, (**b**) elongation at break, (**c**) compression set, (**d**) hardness.

The mechanical performance of rubber rings that are commonly used as a sealing component is under the spotlight. Therefore, tests are conducted on the related mechanical performance of EPDM before and after modification. Figure 3a–c show the tensile strength, elongation at break, compression set, and hardness of EPDM and the EPDM-ZIF-8 composite; the EPDM-ZIF-8 composite exhibits superior mechanical properties. The tensile strength of EPDM increases from 5.6 MPa to 6.3 MPa, elongation at break rises from 135% to 227%, compression set decreases from 14.1% to 13.1% and hardness improves from 72 HA to 74 HA after modification. The enhancement of mechanical properties is due to the Zn^2+^ and porous coordination structure of ZIF-8, which enhance the interfacial interactions between ZIF-8 particles and the EPDM matrix [18].

**Figure 4:**
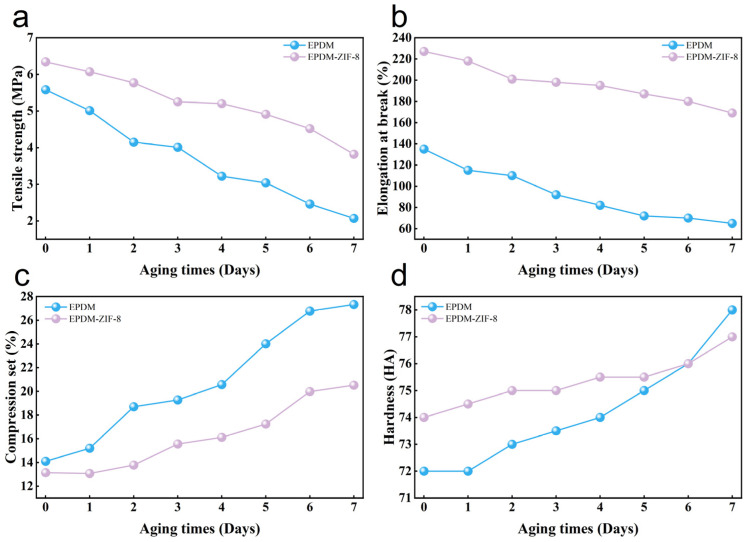
Change in mechanical performance of EPDM and EPDM-ZIF-8 after aging: (**a**) tensile strength, (**b**) elongation at break, (**c**) compression set, (**d**) hardness.

The aging resistance of rubber determines its service life, which is a key concern [24]. To evaluate aging resistance before and after modification, thermal oxygen aging tests are conducted on EPDM and EPDM-ZIF-8. The trends in tensile strength, elongation at break, compression set, and hardness for EPDM and the EPDM-ZIF-8 composite as aging time passes are shown in Figure 4. After thermal oxygen aging for 7 days at 125 °C, the tensile strengths of EPDM and EPDM-ZIF-8 decreased by 63% and 40% (Figure 4a), respectively, and their elongation at break decreased by 52% and 25% (Figure 4b), respectively. In addition, the rate of decline in tensile strength and elongation at break for EPDM is higher than that for the EPDM-ZIF-8 composite. As shown in Figure 4c, the compression set of EPDM and the EPDM-ZIF-8 composite increases with aging time, and the increase in compression set of aged EPDM is 38% greater than that of EPDM-ZIF-8 after aging for 7 days at 125 °C. As displayed in Figure 4d, EPDM exhibits the greatest deterioration after aging for 7 days at 125 °C, with an 8% increase in hardness, while EPDM-ZIF-8 has a 4% increased hardness. In addition, the hardness of EPDM exceeds that of the EPDM-ZIF-8 composite after 6 days. In summary, the EPDM-ZIF-8 composite exhibits superior aging resistance compared to EPDM.

### 3.3. Analysis of Performance Degradation After Aging

The decrease in tensile performance is attributed to the excessive crosslinking density caused by the oxidative crosslinking network resulting from thermal oxidative aging. A moderate increase in crosslink density enhances tensile properties, but excessively high crosslink density leads to a reduction in molecular chain mobility, leading to stress concentration that consequently deteriorates the tensile performance [25]. The compression set of rubber is believed to be the oxidative crosslinked network that is formed during thermal oxidative aging, preventing the recovery of rubber [26]. Therefore, the more oxidative crosslinked networks that are produced by thermal oxidative aging, the higher the compression set is. This increase in rubber in hardness is also due to the growth of the oxidative crosslinked network [27].

### 3.4. Characterization Before Aging

**Figure 5:**
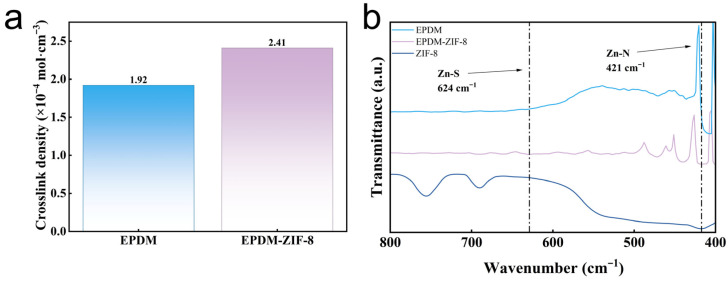
(**a**) Crosslink density before and after modification; (**b**) FTIR spectra before and after modification.

To further investigate the reasons behind the enhanced mechanical properties after rubber modification, the crosslinking densities of EPDM and the EPDM-ZIF-8 composite are measured by the swelling equilibrium method. As shown in Figure 5a, the crosslink density of the modified EPDM increased from 1.92 × 10^−4^ mol·cm^−3^ to 2.41 × 10^−4^ mol·cm^−3^. It is evident that ZIF-8 promoted the crosslinking reaction during the rubber curing process. The enhancement of crosslink density during vulcanization enhances the rubber’s tensile strength, elongation at break, compression set, and hardness; thus, the increase in crosslink density demonstrates that ZIF-8 also bolsters the mechanical performance of rubber by intensifying the crosslinking reaction during the curing process.

The square sodium topology of ZIF-8 endows it with certain adsorption capabilities, enabling it to exhibit excellent sulfur adsorption properties [28]. 2-Methylimidazole, acting as a nucleophile during rubber vulcanization, induces ring-opening reactions in S_8_ [29]. However, the significantly accelerated ring-opening of S_8_ by 2-methylimidazole and excessively high activity of sulfur prevent timely crosslinking between sulfur and the rubber chain after the ring-opening reaction [28]. Uncrosslinked sulfur will be charred within the rubber, leaving impurities that remain in the rubber and negatively impact its properties. The nucleophilic properties of ZIF-8 are due to the effect of 2-methylimidazole. Compared to 2-methylimidazole, the coordination configuration of Zn within the ZIF-8 structure weakens the promoting effect of 2-methylimidazole, allowing sulfur to fully crosslink with the rubber chains [28].

**Figure 6:**
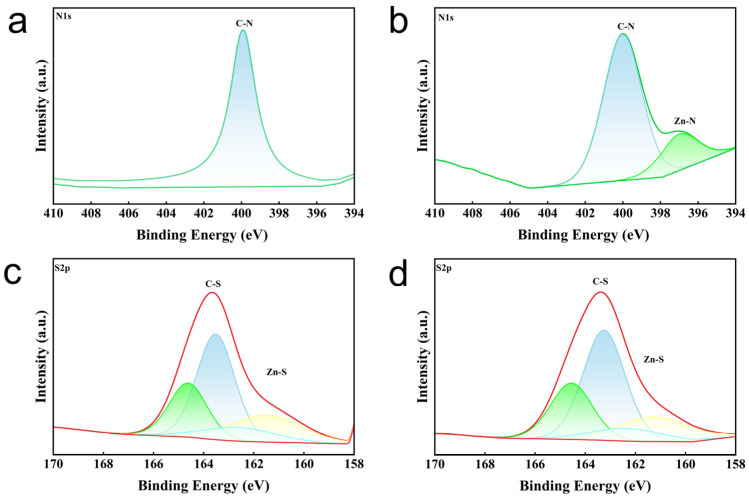
(**a**) N 1s spectra of EPDM, (**b**) N 1s spectra of EPDM-ZIF-8, (**c**) S 2p spectra of EPDM, (**d**) S 2p spectra of EPDM-ZIF-8. In the S 2p spectra, the blue area represents the 2 p_3/2_ peak of C-S, green area represents the 2 p_1/2_ peak of C-S, yellow area represents the 2 p_3/2_ peak of Zn-S, and light blue area represents the 2 p_1/2_ peak of Zn-S.

**Figure 7:**
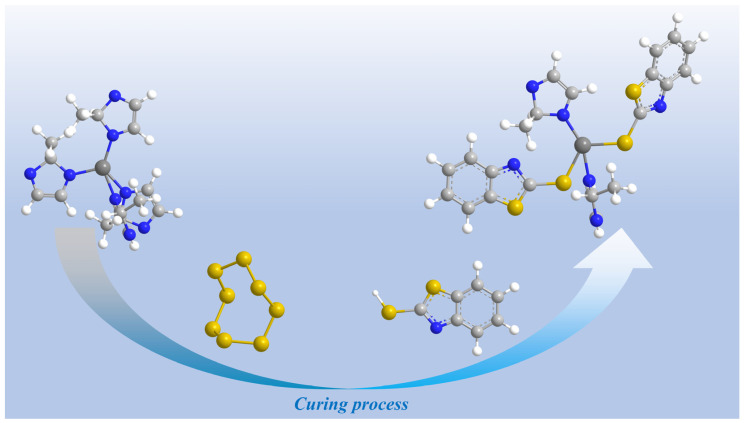
The reaction mechanism of ZIF-8 during the curing process. The blue spheres represent N, the dark-gray spheres represent Zn, the light-gray ones represent C, the white spheres represent H, and the yellow spheres represent S.

To further demonstrate the role of ZIF-8 in the curing process, FTIR and XPS tests are conducted. As shown in Figure 5b, FTIR spectra show Zn-N and Zn-S absorption peaks in the modified EPDM at 421 cm^−1^ and 624 cm^−1^, respectively [30,31]. The Zn-N peak indicates successful incorporation of ZIF-8 into EPDM, because ZIF-8-with-Zn-N bonds are formed after Zn^2+^ coordinates with N in the MIM^−^ ligand (Figure 5b), while the Zn-S peak indicates that Zn forms bonds with sulfur after ring-opening. Moreover, the active sulfide complexes formed by the reaction of zinc with open-chain sulfur promote the curing of rubber [18]. The N1s spectrum of EPDM, as shown in Figure 6a, exhibits only C-N peaks, which is attributed to the presence of the antioxidant 6PPD. The N 1s spectrum of the EPDM-ZIF-8 composite (Figure 6b) exhibits both C-N and Zn-N peaks at 400 eV and 396.5 eV [32,33], indicating successful incorporation of ZIF-8 into EPDM. This result is consistent with the FTIR spectra. As shown in Figure 6c,d, the C-S peak area at 163.5 eV of the EPDM-ZIF-8 composite is larger than that of EPDM [34]. This indicates that ZIF-8 promotes crosslinking reactions during the curing process of rubber, which agrees with the findings in Figure 5a. Additionally, the reaction products of Zn in ZnO with ring-opening sulfur result in the appearance of Zn-S peaks at 161.7 eV in the EPDM S 2p spectra [35]. The reaction mechanism of ZIF-8 during the curing process is illustrated in Figure 7. In summary, the strong interfacial interactions between ZIF-8 particles and the EPDM matrix, as well as the positive effect on crosslinking reactions during the curing process, synergistically enhance the mechanical properties of the modified EPDM.

### 3.5. Characterization After Aging

**Figure 8:**
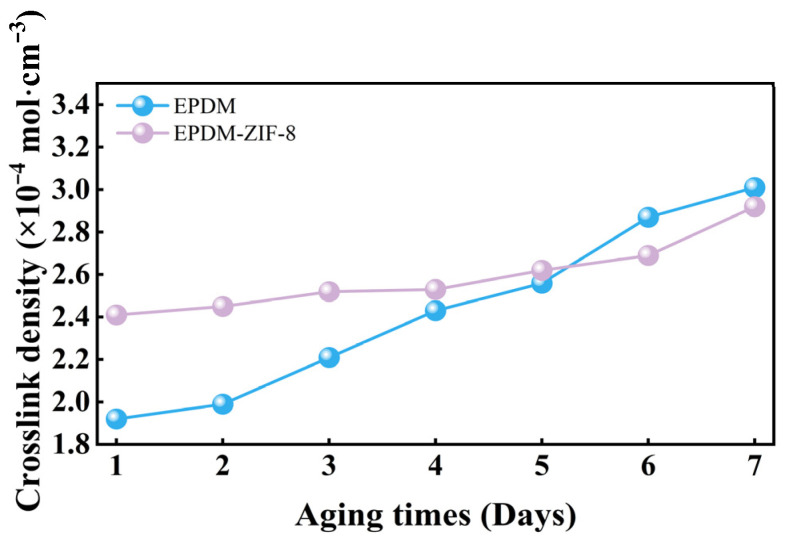
Change in crosslink density of EPDM and EPDM-ZIF-8 after aging.

To investigate the microscopic changes in rubber during thermal oxygen aging, the crosslink densities of two types of rubber after aging are measured using the swelling equilibrium method, and FTIR testing is conducted. In addition, the fracture surface morphologies of both rubbers after aging are observed by SEM testing. The changes in crosslink density over aging time are presented in Figure 8. Both EPDM and the EPDM-ZIF-8 composite exhibit increasing crosslink densities with extended aging durations (Figure 8). EPDM demonstrates a faster rate of crosslink density increase compared to the EPDM-ZIF-8 composite, and its crosslink density surpasses that of the EPDM-ZIF-8 composite after five days. These findings corroborate the mechanical property changes depicted in Figure 5.

**Figure 9:**
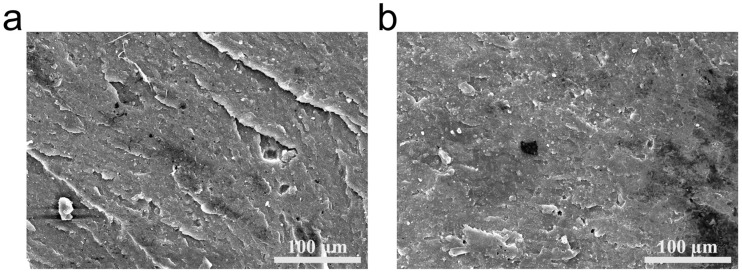
(**a**) SEM images of fracture surface morphology of EPDM-ZIF-8 after aging, (**b**) SEM images of fracture surface morphology of EPDM after aging.

In Figure 9a, the fracture surface of the EPDM-ZIF-8 composite is observed to be densely covered with cracks, while in Figure 9b, the fracture surface of EPDM exhibits fewer cracks. The fracture surface of EPDM is smoother than that of the EPDM-ZIF-8 composite, indicating that the EPDM-ZIF-8 composite exhibits superior aging resistance, as a rougher fracture surface typically signifies better elasticity in rubber [36].

**Figure 10:**
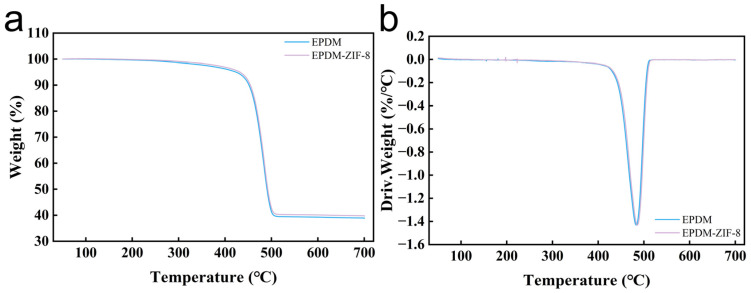
(**a**) TGA images of EPDM and EPDM-ZIF-8. (**b**) DGA images of EPDM and EPDM-ZIF-8.

To thoroughly analyze the effects of different additives on rubber’s aging resistance, TGA testing is conducted on EPDM and the EPDM-ZIF-8 composite. Additive loss is considered to occur at the weight loss point of 200 °C [37]. EPDM exhibits greater weight loss at this temperature, as shown in Figure 10a. Furthermore, Figure 10b demonstrates that EPDM’s weight loss rate is higher than that of the EPDM-ZIF-8 composite. This result demonstrates that ZIF-8 enhances the thermal stability of EPDM, which is credited to the inherently low thermal conductivity of ZIF-8 [38]. In conclusion, ZIF-8 endows superior thermal stability to the rubber, thereby conferring enhanced resistance to thermal oxidative aging to the EPDM-ZIF-8 composite.

## 4. Conclusions

To enhance the mechanical properties and aging resistance of EPDM, this study proposes a straightforward method for preparing ZIF-8 for EPDM modification and analyzes the mechanism by which ZIF-8 influences EPDM. The strong interfacial interaction between ZIF-8 and the EPDM matrix enhances the mechanical properties of the modified EPDM. On the other hand, the nucleophilicity of ZIF-8 promotes sulfur ring-opening during the rubber curing process, which facilitates crosslinking between sulfur and the rubber chains. The Zn coordination configuration of ZIF-8 suppresses side reactions involving sulfur during the curing process, promoting sufficient crosslinking between sulfur and the rubber chains. Therefore, ZIF-8 improves the mechanical properties of EPDM by promoting its curing process, with increases in tensile strength (12%), elongation at break (70%), and hardness (3%, and a decrease in compression set (8%). In addition, the thermal stability of the modified EPDM is enhanced by ZIF-8 due to its low thermal conductivity, which results in the modified EPDM exhibiting less degradation in mechanical properties after aging for 7 days at 125 °C, with improved stability in tensile strength, elongation at break, hardness and compression set.

## Figures and Tables

**Table 1:** Composition of the samples.

Composition (Phr)	EPDM	EPDM-ZIF-8
EPDM	100	100
Silica	40	40
Sulfur	2	2
Steric acid	2	2
MBT	1	1
6PPD	1	1
ZnO	2	0
ZIF-8 capped ZnO	0	2

## Data Availability

The original contributions presented in this study are included in the article. Further inquiries can be directed to the corresponding author.
